# Supplementary material for: Oral Administration System Based on Meloxicam Nanocrystals: Decreased Dose Due to High Bioavailability Attenuates Risk of Gastrointestinal Side Effects
Source: Pharmaceutics. 2020 Apr 1;12(4):313. doi: 10.3390/pharmaceutics12040313 (PMC7238067; doi:10.3390/pharmaceutics12040313)
Supplement: Supplementary file 1 [file pharmaceutics-12-00313-s001.pdf]

pharmaceutics

MDPI

Oral administration System based on Meloxicam Nanocrystals: Decreased Dose Due to High Bioavailability Attenuates Risk of Gastrointestinal Side Effects

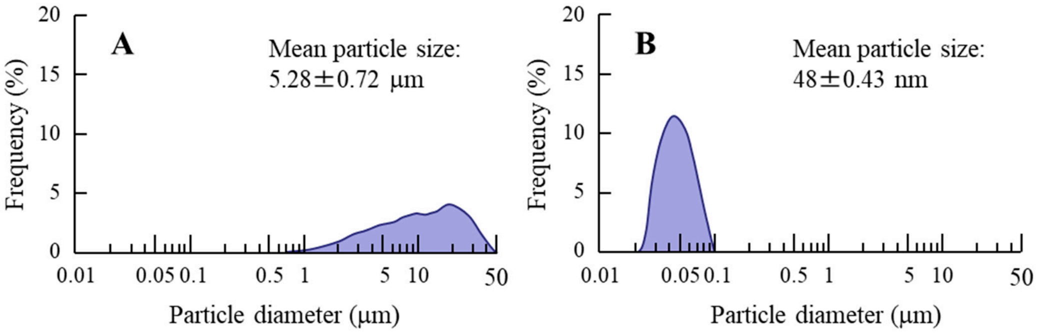

Figure S1. Characteristics of MLX treated with or without the bead mill. **(A)** and **(B)**, particle frequency of MLX-TDs (A) and MLX-NPs (B) by SALD-7100. Refractive index was determined to be 1.60–0.010i (mean ± S.E.).

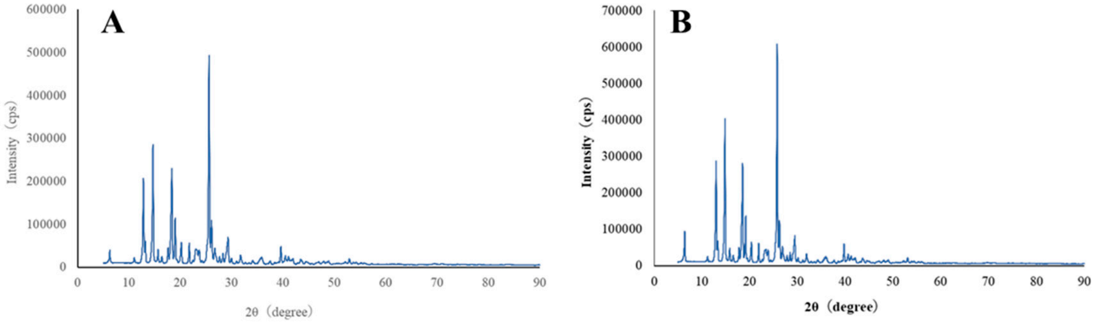

Figure S2. XRD pattern of MLX with (B) or without (A) bead mill treatment. The crystal form was analyzed with a Mini Flex II.

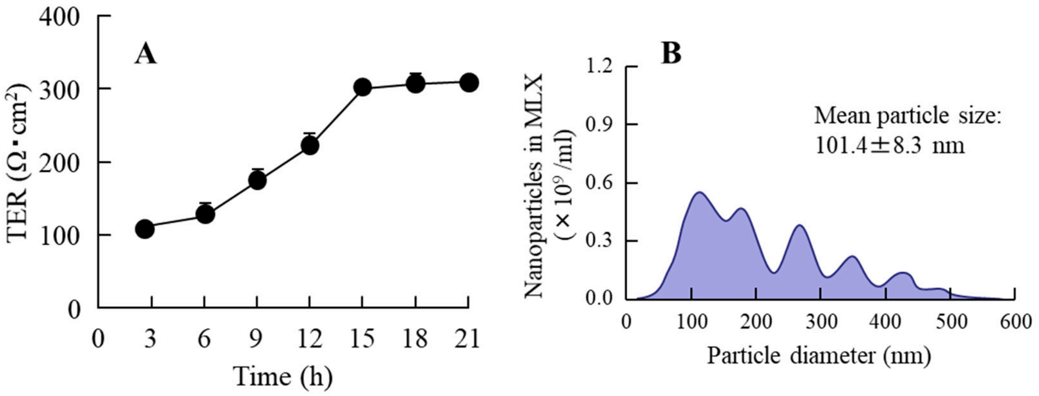

Figure S3. *In vitro* trans-epithelial penetration study using Caco-2 cell monolayers. **(A)**, effect of culture time on TER values of Caco-2 cell monolayers. **(B)**, particle frequency of MLX-NPs on the basolateral side. Particle frequencies were measured by the NANOSIGHT LM10 (mean ± S.E., n=16).

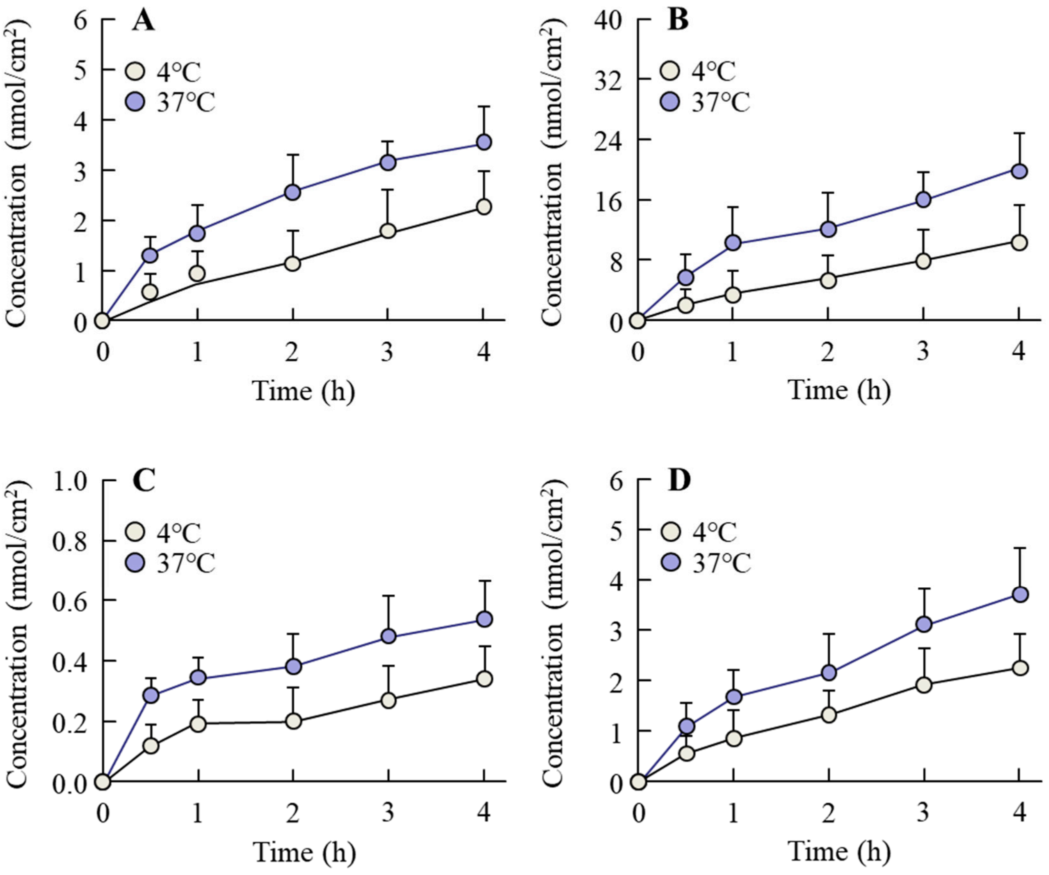

Figure S4. Intestinal penetration of MLX solution and MLX-TDs in the rats. **(A)** and **(B)**, intestinal penetration of MLX through the jejunum (A) and ileum (B) treated with MLX solution at 4℃ and 37 ℃ conditions. **(C)** and **(D)**, intestinal penetration of MLX through the jejunum (C) and ileum (D) treated with MLX-TDs at 4℃ and 37℃ conditions. (mean ± S.E., n=3).

Table S1. Pharmacokinetic analysis of the *in vivo* absorption of MLX formulations.

Formulation	k_a (h^{-1})	t_{max} (h)	C_{max} (μM)	*MRT* (h)
0.2 mg/kg MLX-TDs	1.42 ± 0.29$^{\#}$	4.0 ± 1.0$^{\#}$	0.63 ± 0.13$^{\#}$	12.4 ± 0.84$^{\#}$
0.05 mg/kg MLX-NPs	10.3 ± 0.90*	1.45 ± 0.41*	0.82 ± 0.28	17.8 ± 0.96*
0.2 mg/kg MLX-NPs	10.8 ± 1.1*	1.42 ± 0.49*	3.50± 0.71$^{*,\#}$	19.3 ± 1.02*

The k_e was 2.45×10^{-2} h^{-1}. n=6-10. *P<0.05 vs. 0.2 mg/kg MLX-TDs for each category. $^{\#}P$<0.05 vs. 0.05 mg/kg MLX-NPs for each category.
